# Complications of the anterior retropharyngeal surgical approach to the degenerative cervical spine

**DOI:** 10.1186/1753-6561-6-S4-O3

**Published:** 2012-07-09

**Authors:** S Mihaylova, D Ferdinandov, K Ninov, A Bussarsky, V Karakostov, K Romansky, M Marinov, V Bussarsky

**Affiliations:** 1 Medical University-Sofia, Bulgaria

## Introduction

Anterior cervical retropharyngeal approach for decompression of neural structures is well known and considered as an effective surgical procedure. Opportunities for segment fusion or discoarthroplasty for motion preservation are granted. Both conceptions have their advantages and disadvantages separately from risks of the surgical approach. The aim of this study is to present and analyze the complications of the anterior retropharyngeal surgical approach to the degenerative cervical spine.

## Methods

A database of treated patients at the Clinic of Neurosurgery, Sv. Ivan Rilski Hospital, Sofia, Bulgaria, between January 2006 and August 2011 with cervical spine pathology was used. Certain inclusion and exclusion criteria were applied to select 398 patients with a total of 434 procedures. Comparisons and analysis were done using the observed and recorded initial and follow-up data.

## Results

We present results for intra- and postoperative complications such as dural tears, infections and haemorrhages as well as non-surgically related events. An analysis of risk factors is performed. Risks for adjacent segment degeneration are discussed in details.

## Conclusions

We conclude that the adverse events related to the implant or implantation among groups is not different given the similarity in techniques and treatment course between applied procedures. The most clinically relevant to a patient's quality of life is the adequate decompression of neural structures and expertise based on the patient's individuality.

